# 108 m Underwater Wireless Optical Communication Using a 490 nm Blue VECSEL and an AOM

**DOI:** 10.3390/s24082609

**Published:** 2024-04-19

**Authors:** Ruiyang Tian, Tao Wang, Xiaoyu Shen, Renjiang Zhu, Lidan Jiang, Yongle Lu, Huanyu Lu, Yanrong Song, Peng Zhang

**Affiliations:** 1 College of Physics and Electronic Engineering, Chongqing Normal University, Chongqing 401331, China2022110511027@stu.cqnu.edu.cn (X.S.); 20131121@cqnu.edu.cn (R.Z.);; 2 Department of Electronic Engineering, Chongqing University of Posts and Telecommunications, Chongqing 400065, China; luyl@cqupt.edu.cn; 3 Changchun Institute of Optics, Fine Mechanics and Physics, Chinese Academy of Sciences, Changchun 130033, China; 4 Faculty of Sciences, Beijing University of Technology, Beijing 100124, China; 5 National Center for Applied Mathematics, Chongqing Normal University, Chongqing 401331, China

**Keywords:** underwater wireless optical communication, vertical-external-cavity surface-emitting laser, PPM, AOM

## Abstract

Advanced light sources in the blue-green band are crucial for underwater wireless optical communication (UWOC) systems. Vertical-external-cavity surface-emitting lasers (VECSELs) can produce high output power and good beam quality, making them suitable for UWOC. This paper presents a 108 m distance UWOC based on a 100 mW 490 nm blue VECSEL and an acousto-optic modulator (AOM). The high-quality beam, which is near diffraction-limited, undergoes relatively small optical attenuation when using a conventional avalanche photodiode (APD) as the detector and employing 64-pulse position modulation (PPM). At the time-slot frequency of 50 MHz, the bit error rate (BER) of the UWOC was 2.7 × 10^−5^. This is the first reported AOM-based UWOC system with a transmission distance over 100 m. The estimated maximum transmission distance may be improved to about 180 m by fully utilizing the detection accuracy of the APD according to the measured attenuation coefficient of the blue VECSEL used. This type of UWOC system, composed of a high-beam-quality light source and a conventional detector, make it more closely suited to practical applications.

## 1. Introduction

With the expanding complexity of ocean activities, there is growing demand for high-speed wireless communications underwater. In the area of underwater communications, the primary methods employed include underwater acoustic communications (UACs), underwater radio frequency (RF) communications, and underwater wireless optical communication (UWOC). Among these technologies, acoustic-based UAC has been regarded as the preferable approach to achieve long-distance data transmission at distances of up to tens of kilometers, although its system bandwidth is generally restricted to the kilohertz level, resulting in slower data transmission speeds. As a result, acoustic communication suffers from delays in long-distance transmission, and such delays undermine the real-time character of communication to a certain extent. Acoustic waves are prone to dispersion and reflection during underwater propagation, which leads to multipath effects. Compared with UAC, the propagation speed of signals using RF communication in seawater is many times faster than that of acoustic waves, but the high-frequency signals will be absorbed and attenuated by the water body, which also restricts the long-distance transmission of RF signals in underwater communication [1,2,3]. Compared with the existing underwater communication system, although underwater wireless optical communication is not as good as acoustic wave communication in terms of transmission distance, the optical signal has good directionality, the multipath effect is usually much smaller than in acoustic waves, and its bandwidth advantage can transmit more information in a shorter time [4,5]. The advantages of UWOC can provide more efficient and consistent communication assistance for marine activities. In the future, UWOC will encourage the development of maritime communication technology alongside other communication techniques.

The light source plays a critical role in UWOC systems. Light-emitting diodes (LEDs) and laser diodes (LDs) have been employed as light sources for UWOC systems. LEDs have a wide field of view and are easier to align for transmission and reception [6]. LDs have a small beam-scattering angle to allow for longer transmission links and high transmission rates. Therefore, they are widely used in most high-speed and long-distance UWOC systems [7,8,9]. The common modulation methods in UWOC systems are direct modulation and external modulation. Direct modulation is the direct input of a signal into the driver circuit of a light source, thus changing the output characteristics of the beam. Many studies related to direct modulation have been reported, but due to the limited output power of directly modulated lasers, they have mainly focused on low-power experiments [10,11,12,13]. Acousto-optic modulator (AOM), as an external modulation, has been used in free-space optical communications due to its high extinction ratio, low driving power, and good temperature stability [14]. We believe that an acousto-optic modulator can be used for UWOC to achieve a better modulation bandwidth and a higher optical power, but this would require a more competitive light source [15].

In recent years, there has been substantial improvement in Vertical-external-cavity surface-emitting lasers (VECSELs) technology, principally owing to the adjustable external cavity design that can be modified to satisfy varied needs. Moreover, the performance of VECSELs in terms of producing high output power and good beam quality has received substantial attention from both the academic and industrial sectors [16,17,18]. Consequently, VECSELs can be designed with variable exterior cavities to fit different underwater circumstances, showing tremendous promise for UWOC.

Most UWOC systems use the intensity modulation/direct detection (IM/DD) technique because of the simplicity and low complexity of its implementation. As the distance of communication increases, the signal waveform starts to distort, necessitating the use of increasingly advanced coding and shaping techniques at either the transmitter or the receiver. Compared to on-off keying (OOK), pulse position modulation (PPM) signal formats offer higher power efficiency and better noise immunity for a given bit error rate (BER); therefore, they are widely used in UWOC systems. In 2018, Hu et al. reported a UWOC system using 256 PPM with a transmission rate of 1.7 MHz and a transmission distance of 120 m in Jerlov type II water [19]. In 2021, Yan et al. demonstrated an 84 m/1 Mbps UWOC system using 256 PPM [20]. In 2023, Han et al. demonstrated an underwater wireless optical communication system based on 32 PPM modulation, achieving a communication distance of 35.64 m and a communication rate of 1.9 Mbps [21]. As such, PPM has become the modulation format selected by most researchers.

In this paper, the outer cavity structure of the VECSEL light source is constructed based on the designed gain chip, followed by the measurement of the basic performance parameters of the VECSEL. Then, a 490 nm VECSEL-based UWOC system was designed. The system employed an AOM combined with the PPM to maximize the system’s communication performance. The underwater channel was tested in a 6 m water tank in the laboratory, which was expanded to 108 m by a light reflector. A signal BER of 2.7 × 10^−5^ at the receiver side was obtained for a signal time-slot frequency of 50 MHz, and the baseband signal rate at this time was 4.69 Mbps. This result demonstrates that VECSEL-based and AOM-based systems have good transmission performance in long-distance underwater communication.

## 2. Design of the 490 nm Blue VECSEL

At the core of a VECSEL system is a multi-quantum well (MQW) semiconductor gain chip. In the case of a blue VECSEL, the gain chip is composed of various layers, including the AlGaAs etch stop layer with a high-Al composition, the GaAs protect layer, the AlGaAs window layer with a high barrier, the active region, the distributed Bragg reflector (DBR), and the antioxidant GaAs cap layer that emits light at a wavelength of 980 nm. The active region consists of 15 compression-strained InGaAs quantum wells enclosed by GaAs/GaAsP barriers. The DBR is composed of 30 pairs of alternating high-Al (lower refractive index) and low-Al (higher refractive index) AlGaAs layers, designed to have a center wavelength of 980 nm and a high-reflectance bandwidth of 100 nm, with a reflectivity of 99.9% near the fundamental wave. The VECSEL chips are grown, microfabricated, and diced into small chips with dimensions of 4 mm × 4 mm. The epitaxial end faces of the chips are then treated using titanium-platinum-gold metallization. The chip is soldered and bonded to a copper heat sink, along with a diamond. Subsequently, the GaAs substrate and etch stop layers are removed through a chemical wet etch process, exposing the active region of the chip. The chip assembly is then clamped to a water-cooled copper block for heat dissipation.

The design of the laser-resonant cavity needs to take into account a variety of factors in order to obtain the best laser output effect. Common resonant cavities include straight and folded cavities. Given that the beam waist of a straight cavity is positioned on the chip, adopting a single flat concave mirror is not the most ideal solution for changing the optical field of the external cavity. As a consequence, straight cavities have a poorer frequency efficiency compared to folded cavity configurations. However, the folded cavity creates image dispersion and forms an oval output spot, making the dispersion angle difficult to manage. Consequently, the straight cavity architecture was utilized to ensure that the experimental spot shape remained round. The frequency-doubling crystal (LiB_3_O_5_ (LBO)) within the cavity is positioned as close as feasibly possible to the gain chip without direct contact with the pump light, thereby resulting in a larger power density.

**Figure 1:**
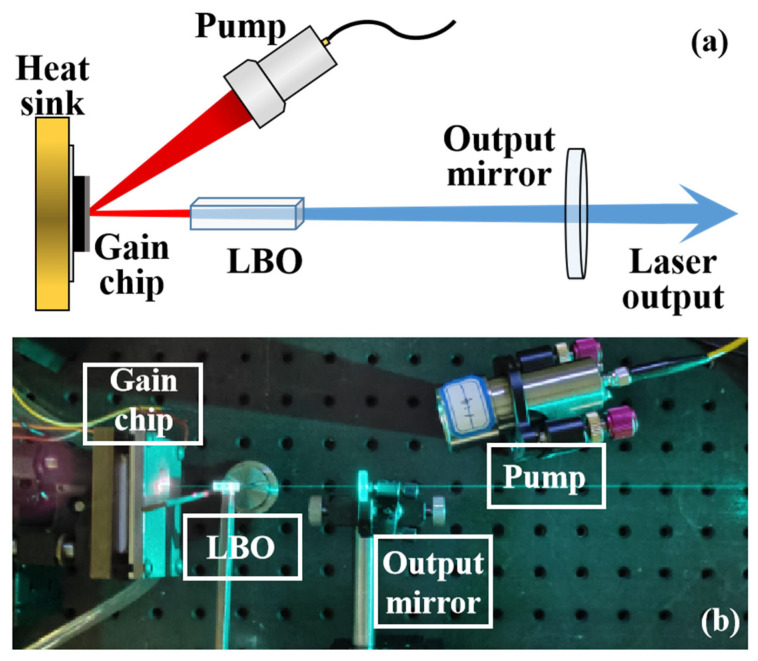
(**a**) Schematic and (**b**) photograph of the 490 nm Vertical-external-cavity surface-emitting laser (VECSEL); The LBO in the figure is the LiB_3_O_5_.

Figure 1 shows the schematic and actual photographs of a 490 nm blue VECSEL. The blue laser used in the experiment has a straight cavity structure. Focusing the 808 nm pump source onto the chip surface through optical fiber coupling, with a 30° incident angle, generates a 980 nm fundamental frequency laser with a beam diameter of 400 μm. A 10 mm LiB_3_O_5_ (LBO) frequency-doubling crystal was positioned within the straight cavity and produced a 490 nm blue laser. The output mirror within the straight cavity is coated to achieve a transmittance of 0.1% at a wavelength of 980 nm and a transmittance of 98% at a wavelength of 490 nm. It is possible to achieve a high level of reflection for a 980 nm laser and a high level of transmittance for a 490 nm laser. The laser has a power conversion rate of 25% from pump light to blue light, and the highest power measured was close to 8 W. However, the experimental setup utilized a laser power of 100 mW, which considered factors such as the transmission distance of the laboratory tank, the detection threshold of the detector, the power consumption of the laser, and the thermal management. This decision not only met the requirements for communication performance, but also guaranteed the stability of the system and its long-term reliability.

**Figure 2:**
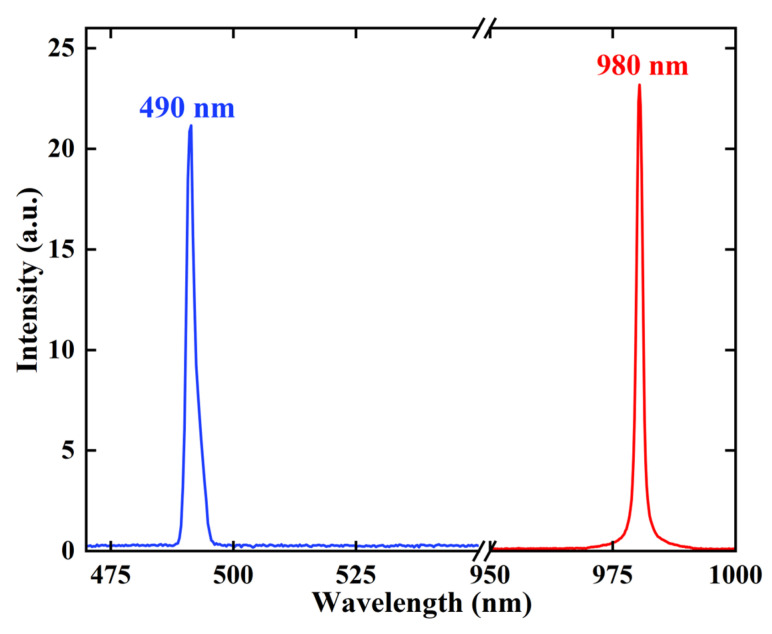
Spectra of the frequency-doubled (**left**) and fundamental (**right**) VECSEL.

**Figure 3:**
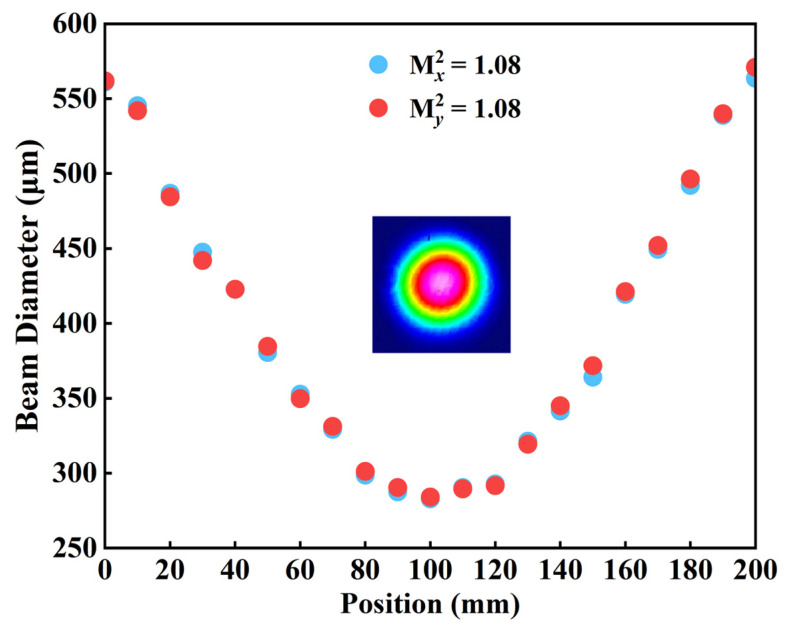
The measured M^2^ factor of the VECSEL.

After constructing the optical cavity, the spectra and beam quality of the VECSEL were measured individually. Figure 2 illustrates the fundamental and frequency doubling light spectra of VECSEL measured using a spectrometer (HORIBA iHR320). The beam quality of the VECSEL is shown in Figure 3. The acquired M^2^ factors, both in the horizontal and vertical directions, were 1.08. Additionally, the blue beam profile maintained a circular shape throughout the entire measurement range, signifying the favorable beam quality of the blue VECSEL.

## 3. Experimental Details

**Figure 4:**
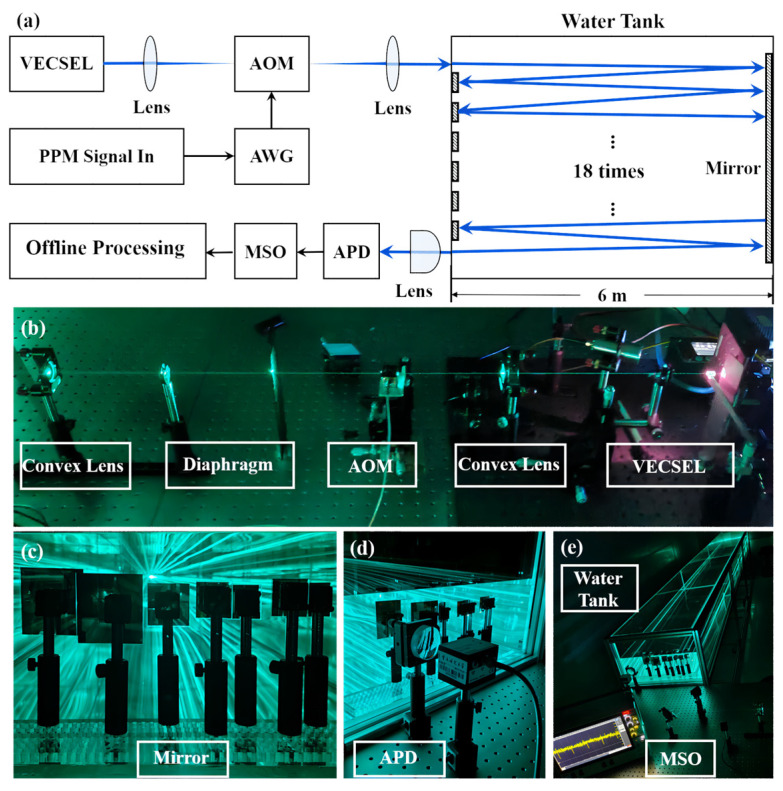
(**a**) Schematic of the 108 m distance UWOC system based on blue VECSEL. AOM in the picture means acousto-optic modulator; AWG in the picture means arbitrary waveform generator; PPM in the picture means pulse position modulation. (**b**) Transmitter of the UWOC system. (**c**) Underwater reflector. (**d**) Receiving equipment is avalanche photodiode (APD). (**e**) Underwater communication link and mixed-signal oscilloscope (MSO).

Figure 4a presents the schematic of the experimental setup for VECSEL-based underwater optical wireless communications using AOM and PPM modulation. Due to the laser’s structure, external modulation was used in this experiment. The UWOC system was first evaluated at different received optical powers, and then tested over the 108 m long distance. The major components of the experimental setup are indicated in Figure 4b–e. All studies were conducted in a dark setting to reduce the effect of optical noise. At the transmitter side, a pseudo-random binary sequence (PRBS) generated by a Personal Computer (PC) was loaded into an arbitrary waveform generator (Tektronix AWG70002A) for digital-to-analog conversion, and the analog signals were transmitted to the driver of the AOM (250 MHz bandwidth) to perform analog modulation of the incident laser beams. A lens with a short focal length (ƒ_1_ = 100 mm) was used to focus the 490 nm blue laser before it entered the AOM. The angle of incident light was adjusted to achieve the optimum diffraction intensity of the AOM, and then an aperture was used to filter out the diffracted light emitted from the AOM. Another lens with a long focal length (ƒ_2_ = 300 mm) was used on the other side to collimate the laser beam to reduce its divergence. The modulated laser beam passed through a 6 m long water tank. The water channel was filled with tap water. To obtain a maximum communication distance of 108 m, reflectors were put on both sides of the water tank. On the receiver side, the output laser beam was focused by a lens onto an avalanche photodetector (Thorlabs APD210), and the APD module was used to convert the received optical signals into electrical signals. A mixed-signal oscilloscope (Tektronix MSO68B) was used for data acquisition, and then the signals were processed offline.

## 4. Results and Discussion

The techniques of modulation and demodulation play a crucial role in the functioning of UWOC systems.

### 4.1. Acousto-Optic Modulator Bandwidth Assessment

**Figure 5:**
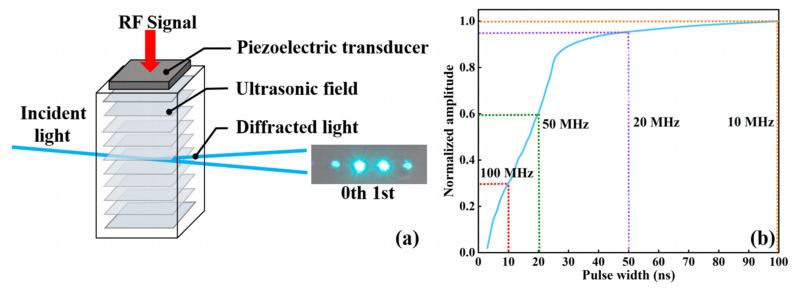
(**a**) Schematic of acousto-optic Bragg modulation. RF in the picture means radio frequency. (**b**) Normalized signal modulation depth corresponding to pulse width.

The external modulation of laser beams uses the Bragg diffraction principle of acousto-optic crystals. As shown in Figure 5a, when the radio frequency (RF) signal is applied to the piezoelectric transducer, it is transformed into an ultrasonic signal and transmitted to the acousto-optic crystal. The ultrasonic wave induces periodic fluctuations in the refractive index of the acousto-optic crystal, resulting in the formation of a phase grating. When the incident light enters the acousto-optic crystal, the angle of incidence determines the diffracted light, and the acousto-optic crystal produces first-order diffraction, carrying the signal only if the Bragg diffraction angle is satisfied. Acousto-optic modulators can achieve diffraction efficiencies of up to 70%.

**Figure 6:**
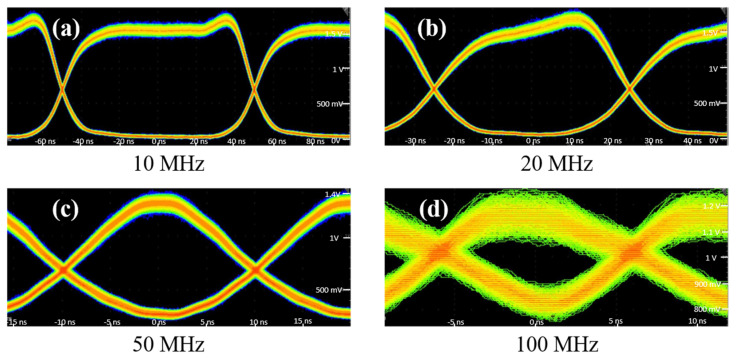
Eye diagrams of signals with frequencies of 10 MHz (**a**), 20 MHz (**b**), 50 MHz (**c**), and 100 MHz (**d**).

The ultrasonic carrier frequency of the AOM was 250 MHz. Considering the bandwidth limitation of the AOM, we measured the modulation of the optical signal by the AOM. As shown in Figure 5b, the signal modulation depth at 10 MHz bandwidth was normalized to measure the modulation depth at different signal pulse lengths. The signal modulation depth did not change much at a pulse width of 50 ns, corresponding to a time slot frequency of 20 MHz. However, at 100 MHz, the modulation depth was only 30% of that at 10 MHz. The corresponding eye diagrams at different frequencies are shown in Figure 6a–d.

### 4.2. Comparison of Modulation

**Figure 7:**
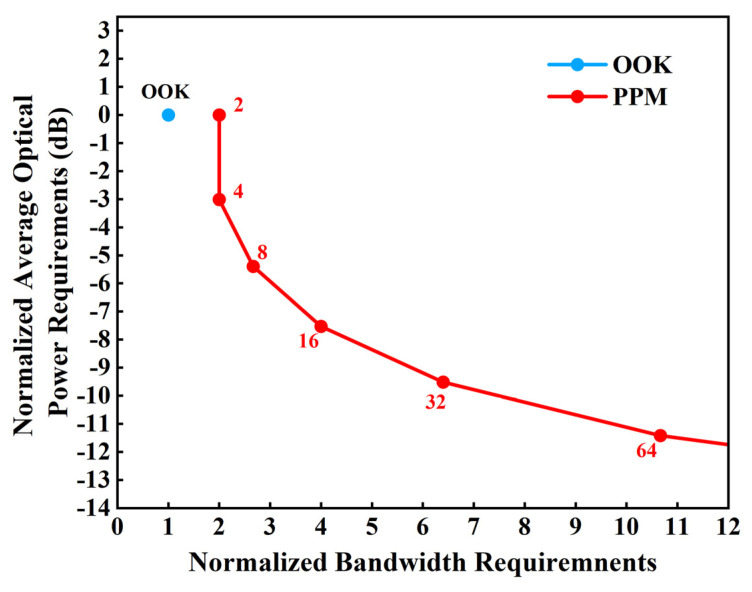
Power requirements versus bandwidth requirements with on-off keying (OOK) and pulse position modulation (PPM).

It is vital to prioritize modulation schemes with high transmission rates or high transmission efficiency while assuring dependability [21,22,23]. Most UWOC studies have been carried out utilizing PPM, or on-off keying (OOK), as the modulation format in the water channel. OOK and PPM are basic intensity modulation methods that are frequently employed in long-distance UWOC systems. OOK modulation is a switch-keyed modulation that performs signal transmission by switching the carrier amplitude. PPM modulation utilizes pulse position modulation to achieve signal transmission by changing the position of the carrier pulse. When the PPM system is in operation, the laser can function at high peak power and low average power, resulting in improved transmission efficiency. Due to the complexity of the underwater environment, different modulation methods have their own advantages. As depicted in Figure 7, the y-axis represents the optical power requirement normalized to OOK, and the x-axis represents the ratio of bandwidth required for PPM and OOK after normalization. The number indicates the number of PPM time slots. As the modulation order increases, the bandwidth requirement of the PPM gradually increases, but the power efficiency also increases. The OOK structure is comparatively uncomplicated and possesses a higher bandwidth efficiency compared to the PPM modulation for transmitting the same communication capacity. Nevertheless, it also requires a higher average optical power.

**Figure 8:**
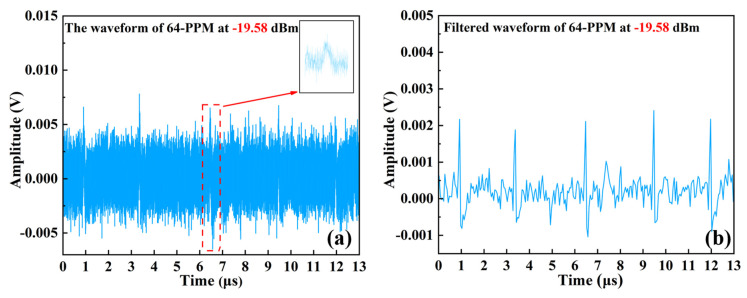
(**a**) Unfiltered 64 PPM signal. (**b**) Filtered and sampled 64 PPM signal.

We evaluated the communication performance of the UWOC system at different received optical powers to better understand the difference between OOK and PPM. The use of hard-decision demodulation in OOK modulation leads to larger-than-expected errors during the demodulation process. The eye diagram of the PPM modulation is already blurred at a received optical power of −19.58 dBm, but since the PPM modulation has no DC component and the PPM pulse width is fixed, it may be easily demodulated by filtering out the noise. As shown in Figure 8a, the signal waveform at the received optical power was −19.58 dBm, and the SNR at this time was 1.79 dB. Figure 8b shows the 64 PPM signal after synchronous equalization and filtering, and its pulse position is clearly visible. This also proves that PPM has better anti-jamming performance compared to OOK modulation.

### 4.3. Measured Performance of UWOC System

To achieve a 108 m VECSEL-based UWOC link, we analyzed the BER performance at different received optical power levels over a specific transmission distance. We varied the time-slot frequency of the UWOC system from 10 MHz to 20 MHz, 50 MHz, and 100 MHz. The received optical power was attenuated at the receiving end using a series of neutral density filters. As the power decreased, the signal attenuation in the UWOC link became more pronounced. Thus, the study of BER versus received optical power is an important method for evaluating the minimum received optical power required for a VECSEL-based UWOC system to achieve a certain data rate over a certain transmission distance.

**Figure 9:**
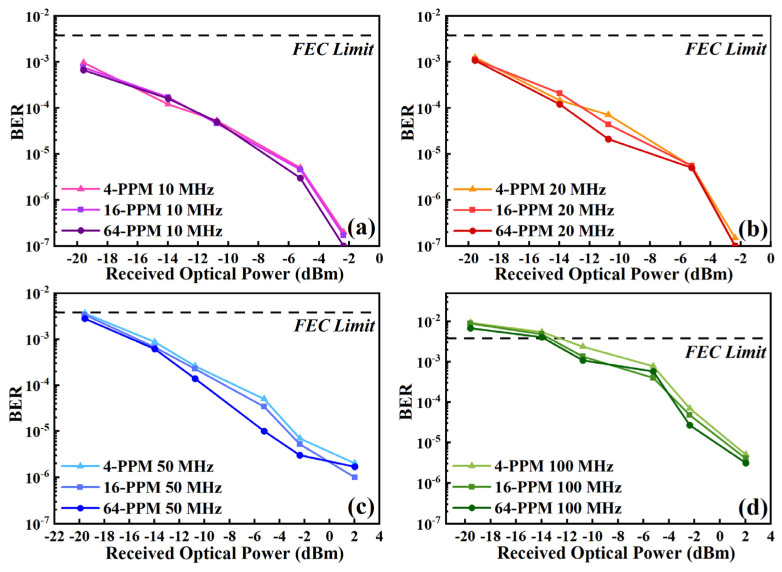
Relationship between bit error rate (BER) and received optical power for different orders of PPM at 10 MHz (**a**), 20 MHz (**b**), 50 MHz (**c**), and 100 MHz (**d**) time slot frequencies; FEC means forward error correction.

Signals of different power levels were recorded at 60 m. The waveform recorded by the MSO illustrates the BER of PPM transmissions at different frequencies. The difference in communication performance between different PPM modulation orders and time slot frequencies were verified by varying the amount of received optical power. Figure 9a–d shows the BER of PPM modulation with different modulation orders at the time slot frequencies of 10 MHz, 20 MHz, 50 MHz, and 100 MHz. Respectively, the BER of the three modulation orders are quite close to each other under the same time-slot frequency. Following analysis, the BER of the 64 PPM was lower than that of the 4 PPM and the 16 PPM, corroborating our prior conclusions. Comparing the BER values for the three modulation orders in Figure 9a,b, the 10 MHz and 20 MHz time slot frequencies are approximately equal when the received optical power is the same, with values close to 10^−3^. However, as shown in Figure 9c,d, the BER increases with increasing frequency. For the PPM signals with a time-slot frequency of 50 MHz, the received optical power is −19.58 dBm when approaching the forward error correction (FEC) limit of 3.8 × 10^−3^. At 100 MHz, the BER is already close to 10^−2^. This is due to the decrease in signal modulation depth and the increase in SNR.

We followed up with experiments using 64 PPM modulation and signals with a time-slot frequency of 50 MHz. In UWOC systems, water serves as the channel medium and plays a particularly significant role in determining the extent of the optical attenuation. The attenuation of light in water channels is primarily caused by absorption and scattering phenomena [24]. When light enters an underwater channel, a portion of its power is absorbed by substances such as water, chlorophyll, and humus. Additionally, some of the power is scattered by suspended particles and dissolved salt [25]. The effect of light attenuation in an underwater environment can be described by Beer–Lambert’s law:

*P_i_* = *P_o_* × e^−*cl*^ (1)

$P_{o}$ represents the emitted optical power on the transmitter side before entering the water channel, while *c* denotes the attenuation coefficient and l represents the channel distance.

**Figure 10:**
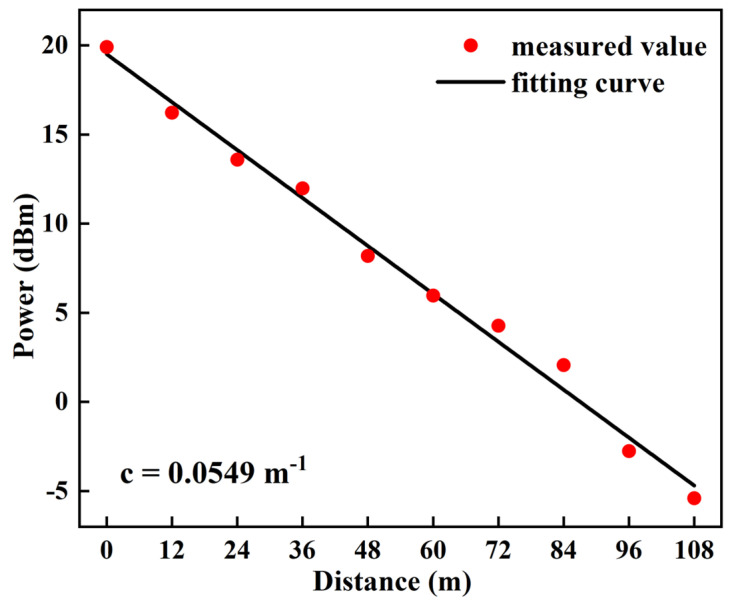
Attenuation coefficient of the used 490 nm blue laser.

Taking the example of a 108 m UWOC channel, we experimented by adding tap water to the tanks, and the attenuation coefficient of the laser was calculated by measuring the received optical power values at different distances. As shown in Figure 10, measurements were taken at intervals of 12 m. Based on the fitting curve, the attenuation of water in this experiment was 0.24 dB/m. Based on Equation (1), the attenuation coefficient can be determined by analyzing the received optical power at two different distances. The attenuation coefficient was therefore calculated to be 0.0549 m^−1^.

**Figure 11:**
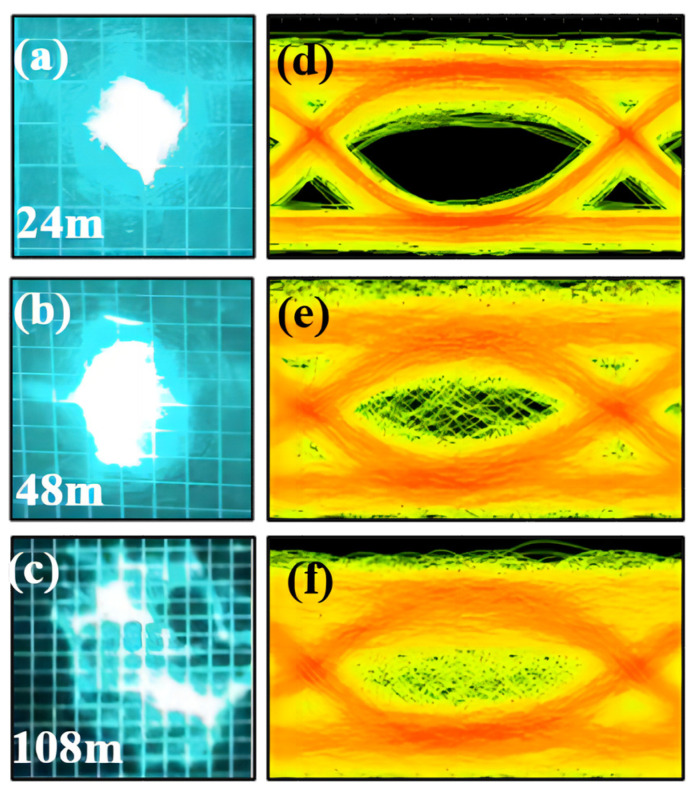
Laser beam size at 24 m (**a**), 48 m (**b**) and 108 m (**c**). Signal eye diagram at 24 m (**d**), 48 m (**e**) and 108 m (**f**).

We tested underwater communication in tap water for a distance of 108 m. We also captured light spot and signal eye diagrams at various distances. As shown in Figure 11a–c, at 24 m and 48 m, the dispersion of the laser spot was still very small. The spot at a distance of 24 m had a diameter of 1.1 cm, and the spot at a distance of 48 m had a diameter of 2.21 cm. The angle of divergence of the VECSEL in water was calculated to be 0.457 mrad (0.0262°). At 108 m, the spot diameter is 6.2 cm, but the laser beam was still focused. This demonstrates the ability of the VECSEL to produce high-quality beams with low divergence angles and high brightness. Observing the eye diagrams in Figure 11d–f, the use of signals at varying distances produced clear eye diagrams.

These findings suggest that the VECSEL exhibits good optical signal stability. Due to the negligible attenuation of the clear water, the optical power received by the receiver was still quite high, despite the distance of 108 m. The VECSEL UWOC system maintained a power of −5.76 dBm at 108 m. The BER performance of a 50 MHz signal with 64 PPM modulation was measured as being at the same distance. The BER at a time-slot frequency of 50 MHz was 2.7 × 10^−5^. This is the first reported AOM-based UWOC system with a transmission distance over 100 m. Based on the optical power results being under FEC constraints and the calculated underwater optical power attenuation coefficients, we could also estimate the predicted maximum communication distance for 64 PPM at 50 MHz to be about 180 m. The size of the spot at the distance of 108 m and the clarity of the eye diagram also suggested that VECSEL-based and AOM-based systems demonstrate excellent transmission results in underwater communications. The channel can still be extended by increasing the optical power, while the small divergence angle of VECSELs and the use of acousto-optic modulation ensure high-quality signal transmission over long distances.

## 5. Conclusions

In conclusion, we demonstrated a 108 m UWOC system utilizing a 490 nm blue VECSEL and an AOM. In this system, the blue VECSEL can provide high-quality laser beams underwater, and the AOM can support the higher optical power of the transmitter. By combining 64 PPM, the anti-interference ability of the UWOC system was improved. Therefore, even using an ordinary APD as the detector, we still achieved a transmission distance of over 100 m and a good BER of 2.7 × 10^−5^ for the communication at the signal time-slot frequency of 50 MHz. This type of UWOC system still presents considerable opportunities to improve communication distances, and acts as a good model for future long-distance UWOC.

## Data Availability

Data are contained within the article.
